# Serum Lipopolysaccharide Binding Protein Levels Predict Severity of Lung Injury and Mortality in Patients with Severe Sepsis

**DOI:** 10.1371/journal.pone.0006818

**Published:** 2009-08-31

**Authors:** Jesús Villar, Lina Pérez-Méndez, Elena Espinosa, Carlos Flores, Jesús Blanco, Arturo Muriel, Santiago Basaldúa, Mercedes Muros, Lluis Blanch, Antonio Artigas, Robert M. Kacmarek, for the GRECIA and GEN-SEP groups

**Affiliations:** 1 CIBER de Enfermedades Respiratorias, Instituto de Salud Carlos III, Madrid, Spain; 2 Multidisciplinary Organ Dysfunction Evaluation Research Network (MODERN), Research Unit, Hospital Universitario Dr. Negrin, Las Palmas de Gran Canaria, Spain; 3 Associate scientist, Keenan Research Center, St. Michael's Hospital, Toronto, Canada; 4 Research Unit, Hospital Universitario NS de Candelaria, Tenerife, Spain; 5 Department of Anesthesiology, Hospital Universitario NS de Candelaria, Tenerife, Spain; 6 Intensive Care Unit, Hospital Universitario Rio Hortega, Valladolid, Spain; 7 Department of Clinical Biochemistry, Hospital Universitario NS de Candelaria, Tenerife, Spain; 8 Critical Care Center, Corporació Sanitaria Parc Taulí, Sabadell, Barcelona, Spain; 9 Department of Respiratory Care, Massachusetts General Hospital, Boston, Massachusetts, United States of America; 10 Department of Anesthesia, Harvard University, Boston, Massachusetts, United States of America; University of Giessen Lung Center, Germany

## Abstract

**Background:**

There is a need for biomarkers insuring identification of septic patients at high-risk for death. We performed a prospective, multicenter, observational study to investigate the time-course of lipopolysaccharide binding protein (LBP) serum levels in patients with severe sepsis and examined whether serial serum levels of LBP could be used as a marker of outcome.

**Methodology/Principal Findings:**

LBP serum levels at study entry, at 48 hours and at day-7 were measured in 180 patients with severe sepsis. Data regarding the nature of infections, disease severity, development of acute lung injury (ALI) and acute respiratory distress syndrome (ARDS), and intensive care unit (ICU) outcome were recorded. LBP serum levels were similar in survivors and non-survivors at study entry (117.4±75.7 µg/mL *vs.* 129.8±71.3 µg/mL, *P* = 0.249) but there were significant differences at 48 hours (77.2±57.0 *vs.* 121.2±73.4 µg/mL, *P*<0.0001) and at day-7 (64.7±45.8 *vs.* 89.7±61.1 µg/ml, p = 0.017). At 48 hours, LBP levels were significantly higher in ARDS patients than in ALI patients (112.5±71.8 µg/ml *vs.* 76.6±55.9 µg/ml, *P* = 0.0001). An increase of LBP levels at 48 hours was associated with higher mortality (odds ratio 3.97; 95%CI: 1.84–8.56; *P*<0.001).

**Conclusions/Significance:**

Serial LBP serum measurements may offer a clinically useful biomarker for identification of patients with severe sepsis having the worst outcomes and the highest probability of developing sepsis-induced ARDS.

## Introduction

Sepsis remains a major challenge in critical care medicine. It is the leading cause of death and its mortality has not decreased substantially in the past decade [1], [2]. An explosion of information regarding the inflammatory response to sepsis has prompted a search for biomarkers that help elucidate molecular pathways that are important in the pathogenesis of the septic process and acute lung injury, that predict outcome, and that may serve as surrogate indicators of potential benefits of therapies [3]–[8]. However, there is not convincing evidence to support the clinical use of any specific marker. Lipopolysaccharide binding protein (LBP), a key participant in the inflammatory response to infection, may be a useful marker for diagnosis and prognosis of patients with bacterial infections [9]. LBP is a type I acute phase response protein that is produced by hepatocytes, respiratory epithelial cells and a myriad of other cell types [10], and enhances the recognition of endotoxin and pathogens by the immune system [11]. LBP binds to Gram-negative bacteria via the lipid A part of the lipopolysaccharide (LPS) which mediates its binding to the CD14 cellular receptor molecule presented by monocytes and macrophages [12], [13]. Binding of LPS activates monocyte/macrophage system cells via Toll-like receptors [14], resulting in the production of pro-inflammatory cytokines that aggravate the clinical presentation of sepsis. In humans, LBP is constitutively present at a mean serum concentration of 5–20 µg/mL [15], [16], reaching peak levels higher than 200 µg/mL during the acute-phase reaction [5], [13], [17]. LBP also mediates the immune response to diverse pathogens including Gram-positive bacteria [18]. Thus, several studies have reported increased LBP serum levels in adult patients with sepsis caused by bacterial and fungal infections [13], [16]–[22]. However, some of those studies have shown conflicting results and others failed to show any correlation between blood levels and disease severity, most likely due to the limited number of patients with severe sepsis [13], [6]–[22] and/or the absence of follow-up LBP measurements [16], [17], [19]–[21].

In this study, using a large, multicenter cohort of patients meeting the international criteria for severe sepsis, we hypothesized that the pattern of LBP serum levels during the first week of severe sepsis development is a marker of severity and prognosis. The goals of this study were to determine: (i) whether there is a different pattern of LBP serum levels between survivors and non-survivors; (ii) the utility of monitoring LBP serum changes within the first 7 days of severe sepsis as a predictor of outcome, and (iii) whether LBP serum levels differ between patients who developed different degrees of lung injury.

## Materials and Methods

### Objectives

We analyzed the time-course of LBP serum levels in patients with severe sepsis to investigate the hypothesis that serial serum levels of LBP could be used as a marker of severity and outcome in patients with severe sepsis.

### Ethics

The study was approved by the Ethics Committees for Clinical Research of the Hospital Universitario NS de Candelaria in Tenerife, Spain, and of the Hospital Universitario Río Hortega in Valladolid, Spain. Written informed consent was obtained from each patient or an appropriate proxy.

### Participants

This multicenter, prospective, observational study included consecutive patients older than 18 years old fulfilling the International Sepsis Criteria for severe sepsis [23] admitted between April 2003 and March 2006 into a network of Spanish intensive care units (ICUs) (see appendix for a list of participating centers). We chose to study only patients with severe sepsis to guarantee severity of illness and a high risk for death. All patients were screened for severe sepsis on ICU admission and daily thereafter. Severe sepsis was defined as sepsis complicated by organ dysfunction [23]. We considered sepsis as a clinical syndrome defined by the presence of both infection and a systemic inflammatory response [23], [24]. Sepsis was microbiologically documented or clinically suspected as a result of the presence of white blood cells in a normally sterile body fluid, a perforated viscus, chest X-ray consistent with pneumonia and associated with purulent tracheal secretion, or other clinical syndromes associated with a high probability of infection. Clinically documented infection was defined by the presence of gross pus or an abscess, but no microbiological confirmation because of ongoing antibiotic therapy. Patients were enrolled into this study within the first 24 hours of meeting criteria for severe sepsis. Patients in whom decisions to withhold or withdraw life sustaining treatment were established within the first 24 hours of ICU admission, were excluded. All patients were screened on a daily basis for the presence of clinical and analytical signs of sepsis and, when indicated, cultures, biopsy or aspiration of the potentially infected sites were obtained. Patients were followed until ICU discharge or death.

As a general approach, and for the purpose of this prospective, observational, multicenter, cohort study, all participating physicians were urged to administer broad-spectrum antimicrobial agents in a timely manner, ensure early identification of causative microorganism, intravenous antibiotics as soon as sepsis was suspected or recognized, and to optimize antibiotic selection and timely administration on the basis of the antibiogram. For the ventilatory management, a tidal volume of 5–9 mL/kg predicted body weight at a ventilatory rate to maintain adequate PaCO_2_, and with PEEP and FiO_2_ combinations to maintain PaO_2_>60 mmHg or SpO_2_>90% were recommended. Fluid resuscitation and vasopressor administration were individualized. The goal was to maintain a systolic blood pressure ≥90 mmHg or a mean arterial pressure of ≥65 mmHg. It was recommended to maintain hemoglobin between 7–10 g/dl [25]. None of the patients in this cohort received activated protein C or low doses of corticosteroids as an adjunctive treatment.

### Data collection

All data were collected on standardized forms by the clinicians responsible for the study in each ICU. Data collection included demographics, diagnoses, comorbidities, source of sepsis and isolated pathogens. Clinical and laboratory data needed to calculate the Acute Physiology and Chronic Health Evaluation (APACHE) II score were collected within the first 24 hours after ICU admission [26]. All patients enrolled in the present study were followed prospectively for the development of acute lung injury (ALI) and acute respiratory distress syndrome (ARDS), as defined by the American-European Consensus Conference [27]. In addition, number of organ failures included in the Sequential Organ Failure Assessment (SOFA) scale [28], date and time of inclusion into the study (fulfilled the criteria for severe sepsis), ICU and hospital length of stay, and ICU, hospital and 28-day mortality were recorded.

### Blood sampling and laboratory analyses

Blood samples (5 mL) were collected within 24 hours of meeting severe sepsis criteria (baseline). Additional blood samples were obtained at 48 hours and at 7 days after study entry, only if the patient remained hospitalized into the ICU. Samples were centrifuged at 4°C for 10 min at 3200 rpm within 35 min after sampling. Three aliquots of serum were collected in cryovials and frozen and stored at −80°C. Participating centers shipped the tubes on dry ice to the research laboratory of the coordinating center. Serum LBP was assayed using a commercially available chemoluminescence fully automated immunoassay in an Immulite 1000 analyzer (Diagnostics Products Corporation, Siemens Medical Solutions, Germany). As stated by the manufacturer, the lower assay limit for the LBP assay was 0.2 µg/ml, and the calibration ranged was up to 200 µg/ml, having a 10^3^ linear dynamic range. The described manufacturer within-run intra-sample coefficient of variation ranged from uppermost values of 5,8% for mean concentrations of 14 µg/ml to 3.3% for a concentration of 43 µg/ml. A dilution series was performed for those samples with initial values above the range limit. Single LBP serum measurements in each patient were determined by the same investigator (MM) who was blinded to the clinical parameters and outcome of patients.

We chose to test several markers within the inflammatory cascade in a subset of 107 patients in whom adequate serum was available. We measured four biomarkers that have pathogenetic basis in sepsis and ALI/ARDS [interleukin (IL)-6, IL-1-beta, IL-10, and C-reactive protein (CRP)] at study entry, at 48 h and at 7 days. Biomarkers levels were measured in duplicate using a commercial kit according to manufacturer's instructions: IL-6, IL-1-beta, and IL-10 (Siemens Medical Solutions Diagnostics, Caernarfon, UK) in an Immulite analyzer, and CRP (Roche Diagnostic, Basilea, Switzerland) in a Hitachi 917 analyzer.

### Statistical analysis

Continuous variables are described as either a mean±standard deviation (SD), or as a median with interquartile range (IQR). Serum levels among different groups were compared by ANOVA or Student t-test. Categorical variables were compared using the Pearson's chi-square test. A logistic regression model with backwards elimination was used to predict disease outcomes by the variation of LBP levels at 48 hours (transformed into a simple 2 status categorical variable: increase or no-increase), adjusting for age, gender, number of organ failures, ARDS and APACHE II score. To compare the evolution of LBP and IL-6, IL-1-beta, IL-10, and RCP levels during the first week, a longitudinal analysis using a general linear model (GLIM) for repeated measures was used to test the variation of markers levels over time within and between groups. A Kaplan-Meier survival analysis was used to compare 28-day survival between patients with and without LBP increase at 48 hours of study enrollment. Finally, to validate and compare the LBP serum levels as a possible biomarker, receiver operator characteristic (ROC) curves and the area under the curves (AUC) were computed to determine the sensitivity/specificity pairs corresponding to particular LBP levels and APACHE II scores for the discrimination between survivors and non-survivors. Data were analyzed by using SPSS 15.0 for windows (SPSS Inc, Chicago, IL). A two-tailed *P* value<0.05 was considered significant.

## Results

We enrolled 180 patients with severe sepsis. Main characteristics of the cohort are presented in Table 1. Mean age was 63.3±15 years and mean APACHE II score was 23.2±6.8. Patients had been hospitalized a median of 1 day (IQR: 0–7 days) prior to ICU admission and 54.4% had severe sepsis on ICU admission. Peritonitis was the leading cause of severe sepsis followed by pneumonia. Blood cultures were negative in 49.5% of cases. Most commonly isolated microorganisms were Gram-negative bacteria. Fifty-five percent developed ARDS and 31.1% developed ALI during hospitalization. Overall ICU mortality was 41.1% (Table 1).

**Table 1:** Main characteristics of 180 patients with severe sepsis.

Variable		Patients (N = 180)
Gender, male/female (%)		59/41
Age (mean±SD)		63±15
Severity (mean±SD)		
	APACHE II score	23.2±6.8
	SOFA score	9.7±3.3
	Number of organ failures	2.3±1.3
	White blood cells at study entry, 10^3^ cells/mL	16,8±9,6
ICU admission		
	Days between hospital and ICU admission, median (p_25_, p_75_)	1 (0–7)
	Days between ICU admission and severe sepsis criteria, mean±SD	1.6±3.1
	Median ICU stay, days (p_25_, p_75_)	7 (3–17)
Identified Pathogen (%)		
	Positive blood cultures	50.5
	Gram-negative only	25.0
	Gram-positive only	15.0
	Fungi only	2.8
	Polymicrobial	7.8
Source of infection (%)		
	Gastro-intestinal tract	47.8
	Respiratory tract	36.1
	Bone and soft tissue	8.9
	Genitourinary tract	4.4
	Catheter related	2.8
Comorbid conditions (%)		
	Insulin-dependent diabetes	7.8
	Immunosuppression	10.0
Lung injury (%)		
	ARDS	55
	ALI	31
Outcome (%)		
	28-day mortality	40.5
	ICU mortality	41.1
	Hospital mortality	46.7

APACHE: Acute Physiology and Chronic Health Evaluation; SOFA: sequential organ failure assessment; ICU: intensive care unit; ARDS: acute respiratory distress syndrome; ALI: acute lung injury.

The mean baseline LBP serum level was 122.5±74 µg/mL (range 26.9–334.0 µg/mL) without significant differences between age, gender, APACHE II score or source of infection. There were no statistical differences of LBP levels in relation to the causative pathogens at study entry: gram-negative infections (136.1±82.8), gram-positive infections (126.4±72.0 µg/ml), fungal infections (96.5±53.7 µg/ml), polymicrobial infections (135.2±73.0 µg/ml) (p = 0.385); at 48 hours: gram-negative infections (91.8±66.3 µg/ml), gram-positive infections (81.7±61.5 µg/ml), fungal infections (62.9±26.3 µg/ml), polymicrobial infections (105.5±63.2 µg/ml) (p = 0.714); and at 7 days: gram-negative infections (76.9±45.8 µg/ml), gram-positive infections (72.7±68.2 µg/ml), fungal infections (40.4±27.9 µg/ml), polymicrobial infections (69.6±49.2 µg/ml) (p = 0.865).Mean LBP levels decreased at 48 hours (93.7±66.8 µg/mL) and at day-7 (73.0±52.4 µg/mL). This decrease was significantly larger in patients who survived and in those who did not develop ARDS. The largest differences in LBP levels were found at 48 hours between patients who developed ARDS and ALI, and between survivors and non-survivors (*P*<0.0001, for both comparisons) (Table 2). Patients developing ARDS had the highest values of LBP.

**Table 2:** LBP serum levels among patients with different degrees of lung injury and in ICU survivors and non-survivors.

Condition	Outcome	Baseline (N = 180)	48 h (N = 147)	7th Day (N = 100)
Lung Injury	ARDS (N = 99)	132.5±76.5	112.5±71.8	79.3±56.1
	ALI (N = 56)	116.4±72.2	76.6±55.9	68.8±46.9
	non ALI/ARDS (N = 25)	96.7±61.9	51.9±30.5	43.0±26.4
	*P*-value*	0.106	<0.0001	0.076
ICU Survival	Survivors (N = 106)	117.4±75.7	77.2±57.0	64.7±45.8
	Non-survivors (N = 74)	129.8±71.3	121.2±73.4	89.7±61.1
	*P*-value‡	0.249	<0.0001	0.017

Values are expressed as mean±SD in µg/mL. LBP: lipopolysaccharide binding protein; ARDS: acute respiratory distress syndrome; ALI: acute lung injury; ICU: intensive care unit.

* *P*- value from one way ANOVA.

‡ *P*- value from *t*-test.

**Figure 1:**
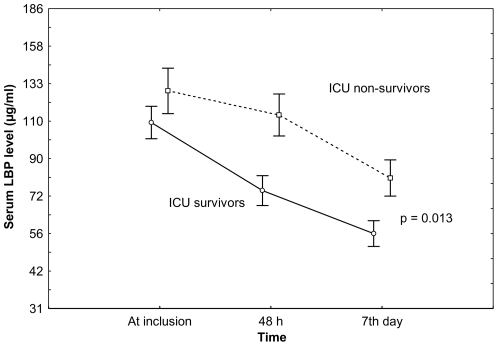
LBP serum levels in 180 patients with severe sepsis during the first week in the ICU. Data are reported as mean (±SE). LPB: lipopolysaccharide-binding protein; ICU: intensive care unit. *P*-value was obtained using a general linear model.

The GLIM test for repeated measures showed that LBP levels during the first week of severe sepsis clearly separated survivors from non-survivors (*P*<0.013) (Figure 1). The initial sample size of 180 patients at study entry was subsequently reduced to 147 patients at 48 hours and to 100 patients after one week due to either ICU death (16 patients at 48 h, 22 patients at day-7), ICU discharge alive (10 patients at 48 h, 26 patients at day-7) and/or non-available samples (7 patients at 48 h, 1 patients at day-7). We noted that patients with an ICU stay less than 48 hours had a higher mortality rate (57.6%, 19 out of 33) than the remaining patients in the study (37.4%, 55 out of 147) (p = 0.033). However, when patients with <48 hours ICU stay were compared to those with ≥48 hours ICU stay, there were no significant differences with regard to age (*P* = 0.457), gender (*P* = 0.205), lung injury (*P* = 0.248), source of infection (*P* = 0.446), or LBP serum levels at study entry (*P* = 0.792).

IL-6, IL-1-beta, IL-10, and CRP levels in survivors and non survivors are summarized in Figure S1 (*see supporting material*). Using the same analyses that we used for LBP, none of those 4 biomarkers showed statistically significant differences among survivors and non-survivors across days examined. Furthermore, since the sample size of the subset of patients in whom we measured these additional markers was smaller (107 vs. 180 for LBP), for each of these markers we generated a simulated 180 patient dataset increasing artificially their sample size by adding measures from 73 randomly selected cases by resampling. Despite this correction, no statistical significant differences were found between survivors and non-survivors. For IL-6 (the marker with best significance level), the significance levels were p = 0.281 at study entry, p = 0.347 at 48 hours, and p = 0.082 at 7 days.

**Figure 2:**
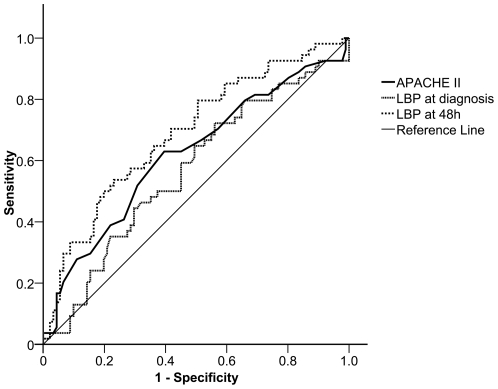
Receiver operator characteristic (ROC) curves for discriminating survivors from non-survivors. Curves were obtained according to lipopolysaccharide-binding protein (LBP) serum levels at study entry and at 48 h. ROC results using APACHE II scores on the day of ICU admission are also plotted for comparison. APACHE: Acute Physiology and Chronic Health Evaluation; ICU: intensive care unit.

ROC curves and AUC (Figure 2) showed that LBP serum levels at 48 hours were a better predictor of outcome than the APACHE II score calculated within the first 24 h after ICU admission. The significance for APACHE II was *P* = 0.016 (AUC: 0.62; 95% CI: 0.52–0.71), for LBP at study entry was *P* = 0.173 (AUC: 0.57; 95% CI: 0.47–0.66) and for LBP at 48 hours was *P*<0.0001 (AUC: 0.71; 95%CI: 0.61–0.80).

**Figure 3:**
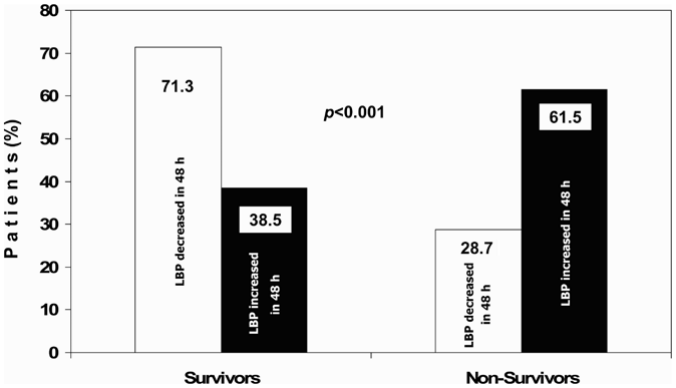
Percentage of survivors and non-survivors patients with severe sepsis according to changes in LBP levels after 48 h of enrolment. LPB: lipopolysaccharide-binding protein. *P*-value was obtained using chi-square test.

**Figure 4:**
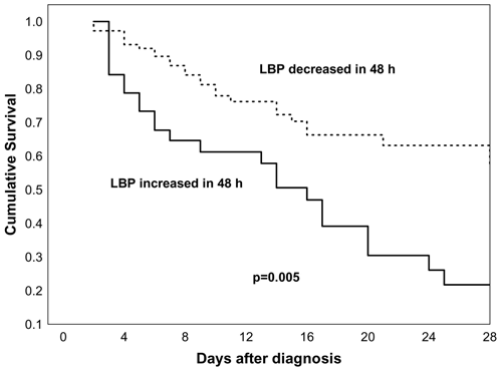
Kaplan-Meier curves for 28-day survival analysis of patients with severe sepsis. In continuous line, patients in which lipopolysaccharide-binding protein (LBP) serum levels increased at 48 h; in discontinuous line, patients in which LBP did not increase at 48 h.

We noted that in 39 patients, LBP serum levels increased at 48 hours compared to baseline (mean increase 49.7 µg/mL) and their mortality was significantly higher than in the 108 patients in whom LBP decreased (mean decrease −52.9 µg/ml) [mortality 61.5% vs. 28.7%, respectively; odds ratio (OR) 3.97; 95% CI: 1.85–8.57, *P*<0.001] (Figure 3). Even after adjusting for age, gender, number of failing organs, and APACHE II score, an increase of LBP at 48 hours continued to predict a higher mortality risk (adjusted OR: 2.76; 95% CI: 1.16–6.54, *P* = 0.018).Kaplan-Meier 28-day survival curves showed strong significant differences between patients with or without increases in LBP levels at 48 hours (log rank test, *P* = 0.0001) (Figure 4).

## Discussion

The main finding of this study is that changes in LBP serum levels at 48 hours after the onset of severe sepsis were associated with disease severity and outcome. Our findings indicate that serial LBP serum measurements may offer a clinically useful biomarker for identification of patients, with severe sepsis, having the worst outcomes and the highest probability of developing sepsis-induced ARDS. In our study, as well as in previous studies [13], [22], serum LBP levels did not differ among patients with gram-negative, gram-positive or fungal infections. The mean LBP serum levels in our series are within the same range as in previous reports [13], [19], [22], [29]–[31].

Blairon et al [20] measured LBP serum levels daily until day 5 or death in a small sample of 24 patients with severe sepsis, but they were unable to demonstrate any correlation between LBP levels and severity, as defined by APACHE II, lung injury and multiple organ dysfunction scores. Prucha et al [13] assessed LBP levels at study entry and at 3- to 5-day intervals for 30 days or until death in a mixed population of 68 patients with systemic inflammatory response, sepsis or septic shock. However, they did not find significant differences in LBP levels between patients with systemic inflammatory response and sepsis or between survivors and non-survivors, since only 9 patients had severe sepsis and/or septic shock. Sakr et al [22] measured the time course of LBP levels in a mixed sample of 327 critically ill patients in which only 55 patients had severe sepsis. As in other previous studies with limited number of patients [13], [16], [30], they found that during the first two days of the disease process LBP concentrations were higher in patients with severe sepsis than in those without sepsis, although no further differences between these groups of patients were observed after the second day. Additionally, Sakr et al [22] concluded that the maximum LBP serum concentration during the first 3 days in the ICU discriminated between survivors and non-survivors, but they were unable to determine if such levels could discriminate between survivors and non survivors among patients with severe sepsis because of the limited sample size (n = 55).

The study by Opal et al [17] is the only published report with a sample size comparable to our study. They measured LBP plasma levels in 253 patients with severe sepsis and/or septic shock and reported mean LBP levels in the lower range of those not only in our series but also in other recent reports [13], [22], [29]. They found that LBP levels were less elevated in non-survivors than survivors. Although the mean APACHE II score in their series was similar to our series, the mortality of their cohort was lower (32.4%) than in our series or that reported in recent epidemiological studies [2], [32]–[35]. Several differences could explain the discrepancies between the two studies. First, the study by Opal et al was a secondary analysis of a population of septic patients selected from the placebo arm of a phase III randomized controlled trial to define the safety and efficacy of recombinant human interleukin 1 receptor antagonist in patients with sepsis [36]. Second, it is plausible that a selection bias occurred since the strict inclusion and exclusion criteria would select patients who were more likely to benefit by the drug under investigation. The observational and non-therapeutic nature of our study design allowed the inclusion of all consecutive patients meeting the criteria for severe sepsis with virtually no exclusion criteria, and therefore, we believe that our patients more closely represent those patients with severe sepsis managed in a routine ICU [35]. The later would explain why our patient population was sicker than the Opal et al. Third, the placebo (control) group in the parent paper by Fisher et al [36] consisted of 302 patients, from which (according to Table 4 in their paper) only 187 patients (62%) had dysfunction of one or more organs, a necessary criteria for being diagnosed as having severe sepsis. However, Opal et al included 253 patients out of those 302 (84%) as having severe sepsis. Forth, although the mean APACHE II score in the Opal et al study was apparently similar to our cohort (26 vs. 23), the magnitude of the standard deviations in both studies (13.6 vs. 6.8, respectively) showed more heterogeneity in their patient population than in our cohort (p = 0.005). Fifth, we do not have much information regarding the prevalence of acute respiratory failure and the use of mechanical ventilation in the Opal study. They reported that 27% of patients had ARDS whereas in our patient population, all patients were mechanically ventilated and 55% had ARDS. We are not surprised by the high prevalence of acute lung dysfunction in our series of severe sepsis since sepsis is the most common clinical condition associated with the development of ARDS and this relationship increases with sepsis severity. In addition, it has been reported that in more than half of patients with severe sepsis without ARDS there is an increased extravascular lung water content, representing subclinical and clinical lung injury [37]. Lastly, there is no enough information on how LBP assays were performed in the Opal et al paper to allow a comparison with our study. Since the data and blood sample collection in the two studies are separated by 15 years, we should acknowledge that differences in patient care, ICU admission policies, the nature of the patient mix, the use of retrospective analysis of data, methods of blood collection and shipping and the technology for LBP assays could explain discrepancies in the reported LBP values.

Our study is the first to report an association between LBP levels and severity of respiratory dysfunction and between LBP levels and outcome in patients with sepsis-induced ARDS. Only 19 ARDS patients were diagnosed during the first 48-hour of study entry; the rest were diagnosed later during ICU hospitalization. Thus, in most cases, the worsening of pulmonary dysfunction followed LBP increase. Martin et al [31] measured LBP concentrations in the bronchoalveolar lavage fluid (BAL) of 82 patients with ARDS. Although they found that LBP increased markedly in the BAL, LBP values were similar in all subgroups of patients and were not related to survival. Cunningham et al [38] assessed time-dependent changes in LBP serum concentrations in 121 trauma patients on hospital admission and at 24 h. In this very low-mortality group (16.3%), a significant increase in LBP concentration was observed at 24 h (28.0±25.3 vs.72.3±45.7 µg/mL). However, although baseline LBP levels were significantly greater in non-survivors than in survivors, after controlling for age and disease severity, LBP concentration did not predict survival. The major difference between those studies [31], [38] and ours is that they measured LBP levels within the first 24 h of admission to the ICU in a heterogeneous population of patients, whereas we performed serial measurements in a population of patients with the same clinical condition (severe sepsis).

In general, our findings and those from other previous reports [13], [16], [17], [19]–[22], [29], [30], [38] support the important role that LBP plays in host-defense during sepsis. In the current study, the variation of LBP concentrations at 48 h predicted worsening acute lung injury and death. We postulate that changes in biomarker levels during the course of severe sepsis may enable physicians to identify those patients who are most at risk for deterioration and who are in greatest need of early intervention. A close examination of changes in LBP serum levels at 48 h and their correlation with outcome enabled us to identify a subgroup of 39 patients (21.7%) in which significantly worse outcomes were observed. In those patients, rather than decreasing LBP levels at 48 h, as expected, the serum concentrations were higher than at onset [Note that these increments were not small (median: 49.7 µg/mL; IQR: 15.1–63.3 µg/mL)]. We cannot explain this finding but can speculate why these patients progressed toward a fatal outcome. During sepsis, LBP levels are modified by polymorphic genetic variation of the *LBP* gene [3], [39]–[41], predisposing these patients to excessive inflammation during an infection and contributing to a poor outcome. Chien et al [41] have recently reported that the presence of a common variant in the 5′ flanking region of the *LBP* gene was correlated with basal LBP serum levels in healthy controls and mortality in patients after allogeneic hematopoietic cell transplantation.

In our study, the changes in the systemic levels of IL-6, IL-1-beta, IL-10, and CRP were unable to discriminate survivors from non survivors during the first week of severe sepsis. In addition, LBP levels at 48 h performed better than APACHE II in predicting ICU outcome. The ROC curve showed that LBP at 48 h dominates APACHE II for any given sensitivity or specificity threshold. The APACHE II requires collection of data regarding numerous variables over a period of time (during the first 24 hours of ICU admission) taking into account the most abnormal values, relies on laboratory data that may be not uniformly collected, and its use is limited by significant inter- and intraobserver variability [42]. It has been reported that several factors influence the performance of severity of illness scoring system: lead time bias [43], case mix, pre-ICU or ICU management, sampling rate of laboratory and hemodynamic data [44], and novel advances in ICU care and therapy since scoring systems were described [26]. On the other hand, it is important to emphasize that the APACHE II score was developed to predict mortality in general ICU population using data during the first 24 hours of ICU stay. On average, patients from our study met severe sepsis criteria almost 2 days after ICU admission and we did not calculate APACHE II at the time patients met criteria for severe sepsis since it is unknown whether the use of APACHE scores generated at the time of patient enrolment result in under or over performance of APACHE II. We speculate that the main reason behind APACHE II not being a good predictor in severe sepsis could be due to APACHE II is a general purpose severity of disease classification system while LBP serum levels might have a more specific association with severe sepsis. Since APACHE II includes many physiological measurements that are not related with sepsis, it is plausible that it does not perform as well on a cohort of patients with severe sepsis in whom the main cause of death (multiple organ dysfunction) is attributed mainly to one factor (severe infection).

It is unlikely that the multifaceted nature of severe sepsis or ARDS would be only monitored with the use of a single biomarker. Microarray-based genome-wide gene expression analysis have shown in animal models that the induction of systemic inflammation by sepsis can cause synergistic effects with acute lung injury in the setting of mechanical ventilation, suggesting that molecules related to the innate immune pathway recognizing the endotoxin might be regulated as well in the presence of lung injury in the absence of infection [45]–[47]. In a recent metaanalysis, Wurfel [48] has indicated that across the different experiments, the most clearly overrepresented theme was “responses to pathogens”, noting that the selected genes for the final analyses were identified as differentially regulated in the presence of injury by mechanical ventilation alone. Given this evidence, and since our study have not been designed to answer the question of whether the LBP response is specific or typical of many acute phase proteins, we suggest that the response is, most likely, non-specific.

There are some limitations to our study. First, confirmatory studies with large sample sizes are required to validate this study. Second, despite the fact that changes in mean LBP levels discriminated survivors from non-survivors, our findings cannot define a cut-off point to clearly identify septic patients at 48 h who will survive from those who will not survive, nor clearly identify septic patients who will develop ARDS from those who will not develop it. Third, a drop off in the number of patients at 48 h (from 180 to 147) could affect the overall significance. However, we do not think that this event would have a major effect on the utility of LBP levels as a prognostic biomarker. It is plausible that those who died within the first 48 h had higher levels of serum LBP in the hours that preceded death, making the overall significance even greater if those values were considered at 48 h. Forth, different therapeutic regimes could influence the LBP values and their associated outcome. However, since it was beyond the scope of this study, we do not know whether treatment-dependent variables may influence the performance of our model under different practice patterns. None of the patients in this cohort received activated protein C or low doses of corticosteroids as an adjunctive treatment. As Kalil et al have recently evaluated [49], the strength of statistical and clinical evidence is weak for most clinical trials on therapies that have been recommended in recent guidelines for treating patients with severe sepsis, particularly for low dose steroids, recombinant human activated protein C, and early goal-directed therapy. Those authors have stated that it is essential to replicate those trials in confirmatory studies before guidelines can be fully adopted by clinicians. In fact, a new PROWESS trial is currently underway to test (and validate) the effects of activated protein C in a high risk septic population.

Our findings add additional justification for evaluating LBP in various high-risk populations during clinical trials. As Minter et al [50] have recently suggested, biomarkers may ultimately serve as targets for future therapeutic trials in severe sepsis whereas it seems essential to choose a biomarker defining appropriate high-risk populations in whom the initial immune inflammatory response is amplified beyond the threshold that is tolerated by the host. Serial monitoring of LBP serum levels may be an appropriate biomarker for subgroup selection and guiding of therapy. We also anticipate that pharmacologic modulation of LBP activation may represent a novel target for future therapeutic trials in the setting of severe sepsis.

In summary, the current study constitutes a step forward over previous published studies investigating the potential use of LBP as a biomarker in sepsis. We have found that a distinct pattern of elevated serum levels of LBP in patients with severe sepsis is strongly associated with increased mortality and the development of ARDS. Additional study is necessary, however, before this or any biomarker can be used to predict outcome in severe sepsis.

## Supporting Information

Figure S1 Comparison of serum levels of IL-6, IL-1-beta, IL-10, and CRP in 107 patients with severe sepsis during the first week in ICU. Data are reported as mean (±SE). IL: interleukin; CRP: C-reactive protein; ICU: intensive care unit. P-value was obtained using GLIM.
(0.49 MB TIF)
