# Supplementary figures and images for: Serum Lipopolysaccharide Binding Protein Levels Predict Severity of Lung Injury and Mortality in Patients with Severe Sepsis
Source: PLoS One. 2009 Aug 31;4(8):e6818. doi: 10.1371/journal.pone.0006818 (PMC2730016; doi:10.1371/journal.pone.0006818)

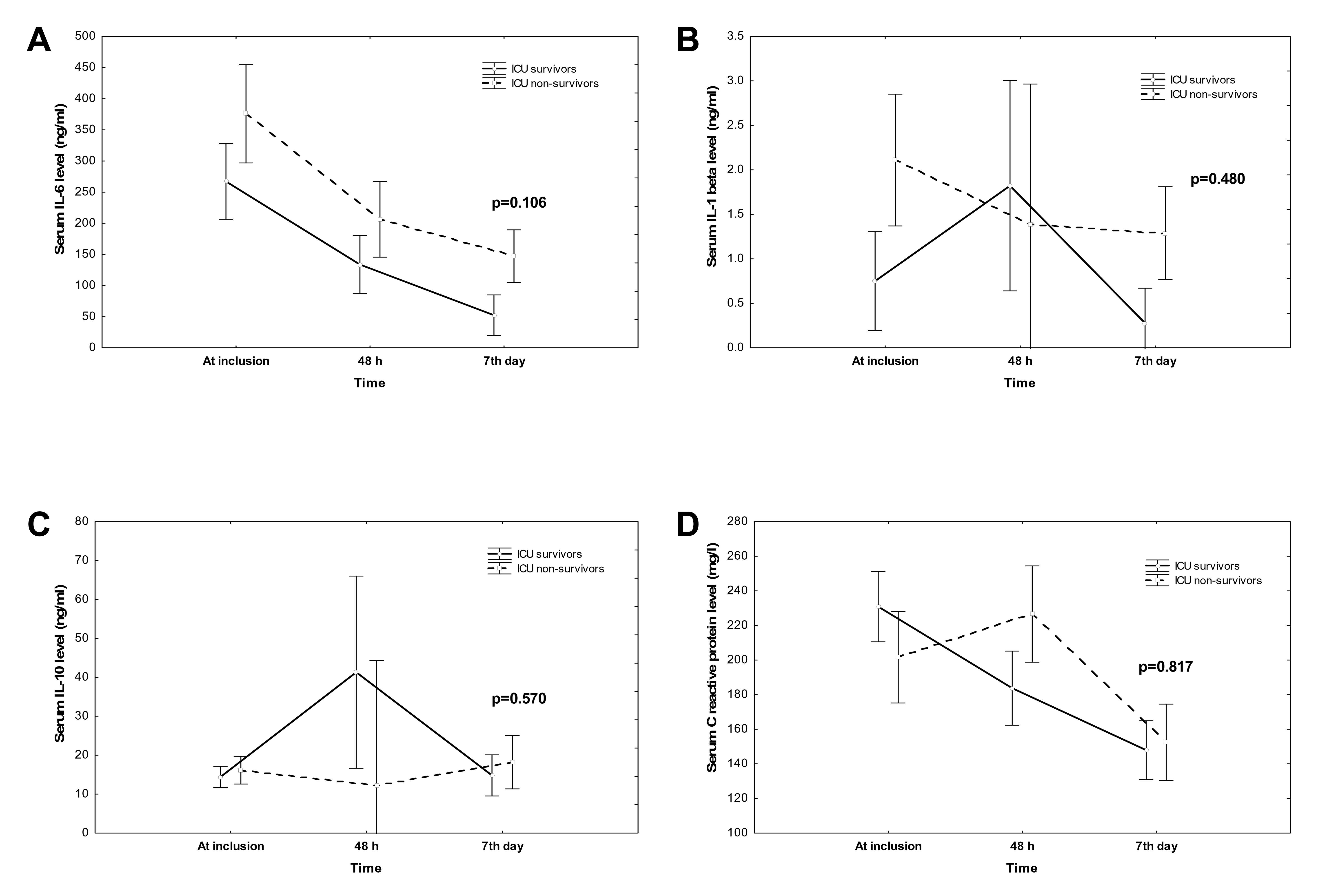

Supplement: Figure S1 — Comparison of serum levels of IL-6, IL-1-beta, IL-10, and CRP in 107 patients with severe sepsis during the first week in ICU. Data are reported as mean (±SE). IL: interleukin; CRP: C-reactive protein; ICU: intensive care unit. P-value was obtained using GLIM. (0.49 MB TIF) [file pone.0006818.s001.tif]
